# Analysis of Factors Affecting the Union of Closed Subtrochanteric Femur Fractures treated by Cephalomedullary Nailing

**DOI:** 10.5704/MOJ.2603.005

**Published:** 2026-03

**Authors:** MN Aroor, MS Kulkarni, S Shetty, S Vijayan, SG Bharadwaj, SK Rao

**Affiliations:** Department of Orthopaedics, Kasturba Medical College-Manipal, Manipal, India

**Keywords:** hip fractures, subtrochanteric fractures, cephalomedullary nailing, malalignment, fracture non-union

## Abstract

**Introduction:**

With surgical advancements and improved implants and instrumentation, nailing is the procedure of choice in subtrochanteric fractures. However, failure in achieving reduction of the multidirectional displacement of the fragments prior to passing the nail, contributes to delayed/non-unions at the fracture site leading to implant failures. In this study, we aim to analyse the factors affecting union of closed subtrochanteric fractures treated by nailing.

**Materials and methods:**

In this retrospective study, closed subtrochanteric fractures treated with cephalomedullary nailing between 2015 and 2019 were included. Demographic, surgical and radiological data were retrieved and analysed. A total of 60 cases were eligible to be included in the study.

**Results:**

Majority of patients were male (50), with a mean age of 46.07±16.40 years. Twenty-two fractures were multifragmentary having a separate butterfly fragment. In 27 patients mini-open technique was used to get the anatomical alignment and to hold reduction until fixation. Overall, the mean time for union was 7.63±5.85 months. We had nine delayed unions and eight non-unions. Varus alignment in the coronal plane of more than 8.5° was the only significant factor associated with delayed or non-union apart from loss of medial continuity.

**Conclusion:**

We recommend achieving fracture reduction with less than 8.5° of varus malalignment in the coronal plane. Varus malalignment is poorly tolerated in fractures at this region. To achieve this, we suggest having a very low threshold to minimally open the fracture site for reduction of these fractures, which does not have any negative effect on the fracture union.

## INTRODUCTION

Subtrochanteric fractures, constituting about 4 – 25% of all the fractures around the hip, occurs in an area with distinct mechanical and biological properties^1,2^. Besides being the area with stress concentrations among the highest in the body, it is mainly composed of cortical bone with critical blood supply^2^.

Although cephalomedullary nails are preferred over extra-medullary devices as the implant of choice in managing subtrochanteric fractures, they are known to have complications like non-union, malunion, loss of reduction, implant failure, infection and associated increase in morbidity and mortality^1-3^. The relative avascular region, medial comminution, higher physiological stresses in this region, poor bone quality, unstable fracture patterns, malreduction in the coronal and sagittal plane, extensive stripping of the periosteum are few of the reasons cited for the occurrence of these complications^1,3^. During nailing, the wide proximal canal further accentuates the malalignment unachieved during reduction. Non-union rates of up to 7% have been reported in cases of malalignment which cause considerable stress transmission across the fixation devices causing failure^4^. It is inevitable to notice that the quality of fracture reduction is much more important than the choice of implant as shown in literature before^5^.

Hence, fracture reduction can be considered to be the most important factor along with preservation of biology and fracture stability in determining the outcome of these fractures. There are not many studies in literature which correlate the effect of these factors on fracture healing. Also, there is no study which quantifies the amount of acceptable reduction to prevent failure. In this study, we aim to analyse the factors affecting the union of subtrochanteric fractures treated by cephalomedullary nailing with special focus on the quality of reduction.

## MATERIALS AND METHODS

This retrospective study was conducted at a level one trauma centre on consecutive closed subtrochanteric fractures who were treated between 2015 and 2019. Institutional ethical committee clearance for this study was obtained. This study has been performed in accordance with the international ethical standards as per the Helsinki declaration.

Skeletally mature patients treated with cephalomedullary nailing and having minimum follow-up of one year or until the fracture union were included in this study. Fractures older than two weeks, treated elsewhere, open fractures and non-osteoporotic pathological fractures were excluded from the study. Demographic data of the patients, mechanism of injury and data regarding the surgical procedures were collected from the patient’s hospital records. Radiographic data was retrieved using the Picture Archiving and Communication System (PACS). Standardised Antero-posterior (AP) and lateral views of the involved femur and AP view of the pelvis with translateral view of the involved hip were taken pre-operatively and at follow-ups. All the fractures were fixed by a single trained senior trauma surgeon with his team.

**Fig. 1:**
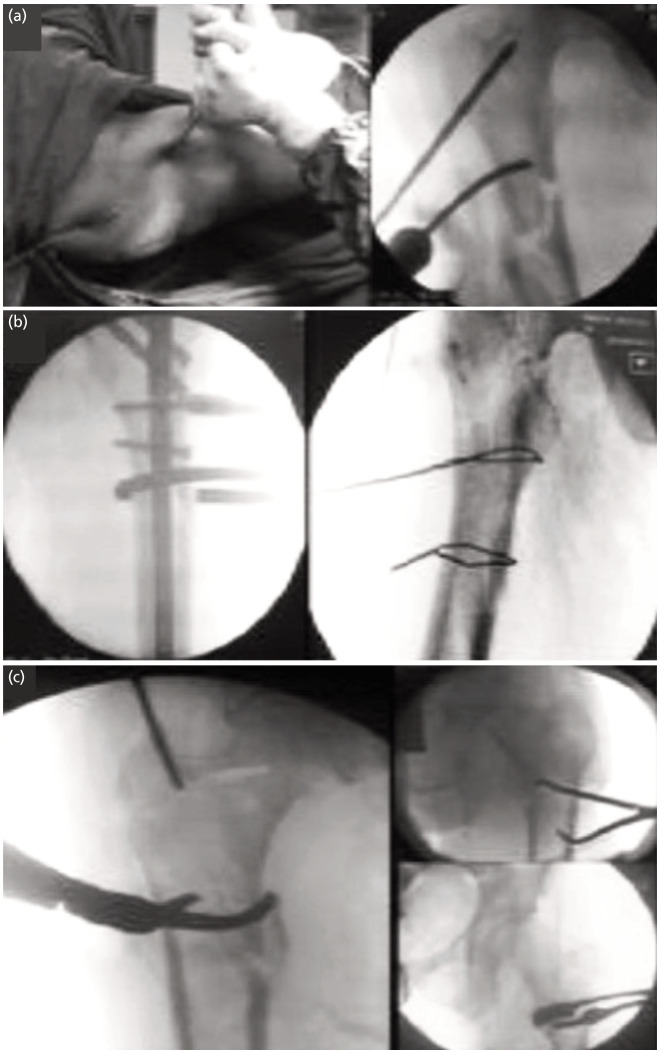
Intra-operative images of mini-open reduction techniques used, (a) Pins used as joysticks to reduce the fracture, (b) collinear clamp and wire loops to reduce fracture, (c) different bone clamps to reduce the fracture.

The patients were positioned supine on a fracture table with traction for fixation. Closed manipulation was attempted in all the fractures to achieve reduction. In case if fracture reduction was not achieved by closed methods, a 2 – 3cm incision was made at the level of the fracture. Ball spike / Schanz pin / Hohmann retractor/ pointed reduction clamp/ bone clamps, collinear clamp or circlage wire was used to reduce the fracture and to hold the reduction provisionally (mini open techniques) as explained earlier in the literature^6,7^. Unicortical Schanz pin was used in the proximal fragment as a joystick to correct the external rotation and abduction along with anterolateral ball spike pusher to correct the flexion, aiding in reduction (Fig. 1a). Prior to the entry point for the nail, fracture reduction was confirmed under image intensifier and was accepted with maximum contact between the major fragments by correcting the rotational and axial displacements. In large butterfly fragments and in long spiral fractures, use of circlage wire, bone holding forceps or collinear clamp was done with minimal damage to the soft tissues (Fig. 1b,1c). Entry point for the nail was made slightly medial to the tip of the greater trochanter^8^. Guide wire was passed, serial reaming was done and cephalomedullary nail inserted. After inserting the neck screw and/or proximal locking screws and distal locking screws sequentially, all reduction instrumentations were removed except the circlage wires. All nails were locked distally in the static mode with two lateral to medial screws. As per standardised mobilisation protocols of the department, all patients were started on static quadriceps strengthening exercises (QSE) on the same day. Dynamic QSE and active straight leg raising exercises were started on post-op day (POD) 1 as per patient tolerance. The patients were then made to weight bear partially and walk on POD1 using walking aids (crutches/walker) as per patients’ tolerance and post-op fitness^9^. Patients were discharged on POD 2 after mobilisation and 2 doses of parenteral antibiotics as per department protocol, if deemed fit. Partial weight bearing was continued till the fracture union. During follow-up, radiographs were taken every six weeks until the fracture union following which patients were reviewed once in three months till a year and six monthly after that to look for complications.

**Fig. 2:**
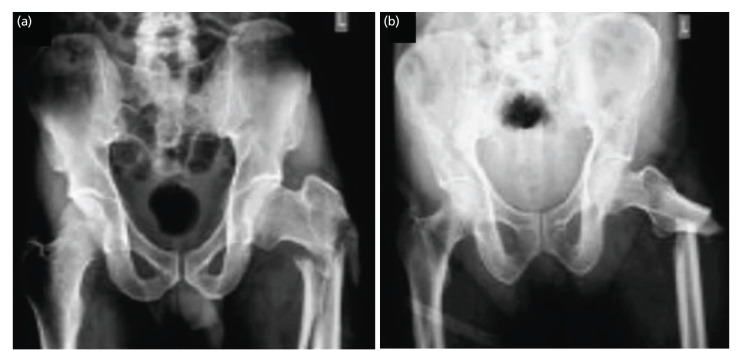
(a) High type of fracture, (b) low type of fracture.

Radiographs were studied for high or low type of fractures, presence of butterfly fragments if any, complications like implant failure or screw cut out. Fractures were classified as high, if the subtrochanteric fracture line was extending into the pyriformis fossa or greater trochanter and as low, if the subtrochanteric fracture was at or below the level of the lesser trochanter having intact greater trochanter with the proximal fragment (Fig. 2). Bone quality was assessed by measuring Cortical index in the lateral radiograph of proximal femur as described by Sah *et al*^10^. Cortical index of ≤0.4 was considered as osteoporosis. Angulation of the proximal fragment with respect to the distal fragment was recorded in both coronal and sagittal planes in the immediate post-op radiographs by two independent observers at two separate occasions. Time taken for union in months was noted. Union was defined as the presence of bridging callus in at least three of the four cortices in anteroposterior and lateral radiographic views. Any fracture which took more than six months for union was categorised as a delayed union^9^. Any fracture which underwent an additional procedure of bone grafting/revision surgery for implant failure/re-fixation was categorised under non-union.

Among the factors affecting the outcome, categorical variables such as gender, affected side, mechanism of injury, associated injuries, comorbid conditions, fracture type, osteoporosis, reduction method, presence of butterfly fragment were analysed using Chi-square test. Student t-test was used to compare the means of continuous variables such as age, cortical index in the lateral proximal femur radiograph, angulation in the coronal and sagittal plane in the post-op radiographs. Time taken to union, union status and additional procedure if any were the outcome variables. Logistic regression was used to calculate the outcome using angulation in the coronal/sagittal plane. Statistical power analysis estimated the sample size to be 54 and with a 10% rate of loss of follow-up, estimated the effective sample size to be 59. Inter and intra-observer reliability were analysed by Interclass correlation coefficient which showed a high degree of reliability (P<0.001). Statistical analysis was done using SPSS software v 20.0 IBM Corporation. A p-value <0.05 was considered significant.

## RESULTS

Of the 76 patients who were treated for subtrochanteric fractures in our institution between 2015 – 2019, nine patients (three had chest injuries, two had fat embolism and four had associated ipsilateral distal femur fractures) were treated using extra medullary devices. Two patients were diagnosed to be having pathological fractures, and five patients were lost for follow-up before fracture union. Hence, the remaining 60 patients were considered for the study.

Of the 60 patients, majority were male (50/60) with involvement of right lower limb more commonly (31/60). High energy trauma (37/60) was the most common cause of injury with mean age of involvement being slightly on the younger side (46.07±16.40 years). Comorbid conditions were present in 14/60 patients. Total of 17 patients had associated injuries (Table I). Out of these, 15 patients had other bone/bones fractures. One patient had ipsilateral tibia, and another had ipsilateral fibula fracture. Four patients had radius fracture, three had clavicle, two had humerus, two had scapula and one each had involvement of femur, bimalleolar, coronoid, calcaneum and phalanx fracture. Three of these patients had more than one fracture apart from subtrochanteric fracture. The mean delay between the admission and fixation was 3.10 ± 2.32 days. Mean period of follow-up was 28.52 ± 6.23 months.

**Table I:** Demographic details, fracture characteristics, fracture healing and complications details of 60 subtrochanteric fractures.

**Variables**	**All patients**
No of patients (Male / Female)	50 / 10
Age	46.07 ± 16.40 years (18 to 85)
Affected limb (Right / Left)	31 / 29
Mechanism of injury (High energy / Low energy)	37 / 23
Associated injuries (Yes / No)	17 / 43
Other Bone fractures	15
Pelvic injuries	5
Chest injury	3
Abdominal injuries	2
Head Injury	1
Comorbid conditions (Yes / No)	14 / 46
Hypertension	10
Diabetes Mellitus	4
Stroke	3
Seizure disorder	1
Myasthenia Gravis	1
Thyroid Carcinoma	1
Retro Viral Disease	1
Stroke	1
Time delay for fixation after admission	3.10 ± 2.32 days (1 to 12)
Fracture pattern (High / Low)	17 / 43
Butterfly Fragment	
Lateral (High / Low)	9 (4 / 5)
Medial (High / Low)	13 (nil / 13)
Nil	38
Reduction Methods	
Closed	33
Mini Open	27
Mean Angulation in AP Radiograph	5.22° ± 5.57° (-7.95° to 17.40°)
Mean Angulation in LAT Radiograph	2.13° ± 5.40° ( -9.86° to 14.70°)
**Union Status**	
Union	45
Delayed Union	9
Non-union	6
Overall Union time	7.63 ± 5.857 (3 to 27 months)
United Fractures	4.84 ± 0.976 (3 to 6 months)
Delayed union	13.22 ± 5.01 (8 to 24 months)
Non-union	20.17 ± 6.43 (12 to 27 months)
High fracture	5.94 ± 3.73 (4 to 20 months)
Low fractures	8.30 ± 6.42 (3 to 27 months)
**Complications**	
Delayed union	9
Non-union	6
Implant failure with non-union	1
Screw Back out with non-union	1

High level of fracture pattern was noted in 17/60. Twenty-two patients of the total study group had a butterfly fragment. Four of the high type (out of 17) fracture pattern were associated with lateral butterfly fragment. In the low type (out of 43), the medial butterfly was more frequent than lateral (Table I). In 27/60 patients, mini open technique was used to achieve the reduction.

**Fig. 3:**
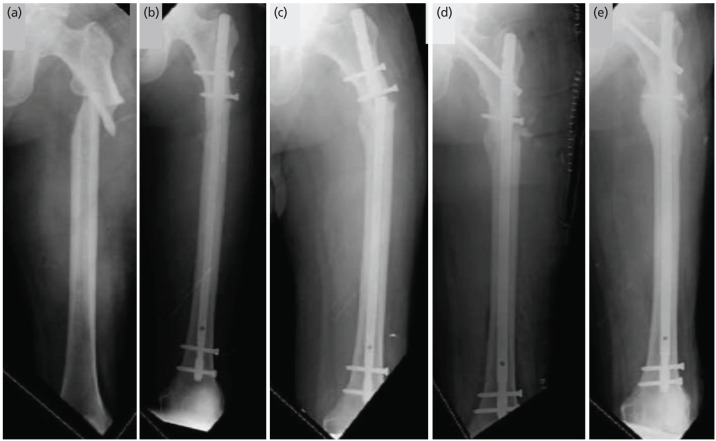
A total of 68-year male with H/O slip and fall with varus malreduction of 10.27°. (a) Pre-op radiographs, (b) post nailing radiograph, (c) implant failure with non-union at 21-month follow-up, (d) revision nailing with bone grafting, (e) united fracture at nine-month post-re-nailing and bone grafting.

Circlage wire was used in two patients to hold the reduction who had low type, long spiral fractures. Overall mean time to union was 7.63 (range: 3-27) months. A total of 45/60 fractures united within 6 months. Six fractures ended up in non-union. All these six patients underwent additional procedures (after ruling out infection). One case of non-union who was not willing for any additional procedure during follow-up, ended up with a broken nail at the non-union level (after 21 months of index procedure) and underwent refixation with thicker nail and bone grafting. It then went in for union after nine months of the procedure (Fig. 3). One case of neck screw back out and subsequent loss of reduction was managed with implant removal and Dynamic Hip Screw fixation with bone grafting and went on for union (Table I). Another case of non-union with thinner nail underwent exchange nailing with bone grafting. The remaining three non-unions (with stable implants in situ) united by bone grafting procedure alone.

**Fig. 4:**
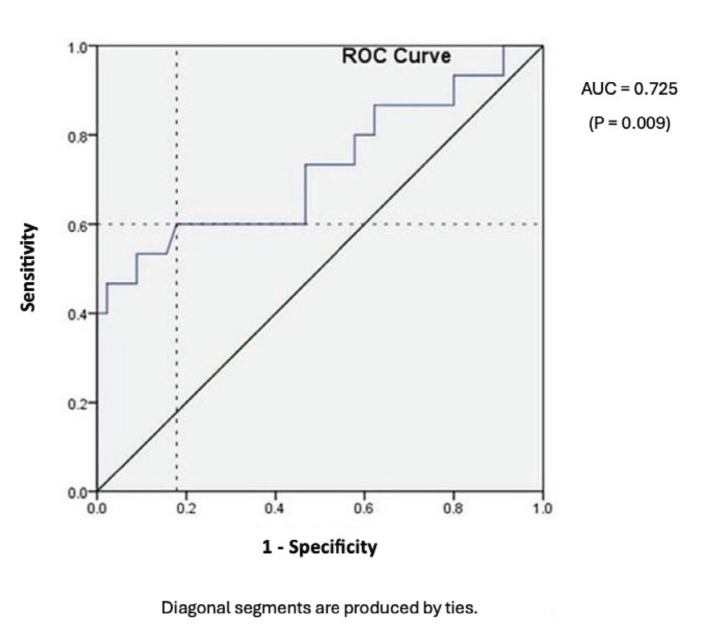
ROC analysis of coronal angulation versus union status showing AUC 0.725 with p<0.05.

There was no significant effect of gender, side involved, mechanism of injury, comorbid conditions, presence of associated injuries and osteoporosis on facture union. But when butterfly fragment was present; the medial butterfly fragment was significantly associated with delayed or non-union (Table II). Mini open reduction of the fracture was not associated with increase in incidence of delayed or non-unions and no infection was noted in any of these fractures. Varus alignment in the coronal plane was significantly associated with delayed or non-union rates but similar association was not found with sagittal plane malalignment (Table II). Receiver Operating Characteristic (ROC) curve analysis of the varus malalignment and union state gave cut off value of 8.5° with significant (p<0.05) Area Under Curve (AUC) which was 0.725 (Fig. 4).

**Table II:** Comparative analysis of fracture union status vs other factors affecting the outcome in subtrochanteric fractures.

**Variable**	**United (45)**	**Delayed / non-union (15)**	p- value*
Gender (Male / Female)	37 / 8	13 / 2	0.689
Affected side (Right / Left)	22 / 23	9 / 6	0.456
Mechanism of injury (High energy / low energy)	27 / 18	10 / 5	0.646
Co-morbid conditions (Yes / No)	10 / 35	4 / 11	0.724
Associated Injuries (Yes / No)	12 / 33	5 / 10	0.620
Osteoporosis (Yes / No)	2 / 33	2 / 13	0.258
Fracture type (High / Low)	16 / 29	1 / 14	0.032 †
Butterfly fragment (Yes / No)	16 / 29	6 / 9	0.757
Butterfly Fragment (Medial / Lateral)	7 / 9	6 / 0	0.017 †
Medial Butterfly (Yes / No)	7 / 29	6 / 9	0.125
Lateral Butterfly (Yes / No)	9 / 29	0 / 9	0.104
Reduction Technique (mini-open / Closed)	28 / 17	5 / 10	0.051
Mean Angulation in AP Radiograph	4.00° (± 4.77°)	8.87 ° (± 6.32°)	0.003 †
Mean Angulation in Lat Radiograph	1.61° (± 5.49°)	3.88 ° (± 5.18°)	0.167
Age in years	44.89 (± 16.88)	49.6 (± 14.84)	0.340
Proximal femoral Cortical Index (Lateral)	0.52 ± 0.069	0.53 ± 0.088	0.65

Notes - * Chi square or Student t test, † : p<0.05

## DISCUSSION

In this retrospective study, we have analysed the factors affecting union in closed subtrochanteric femur fractures. Restoration of the cervico-diaphyseal angle along with correction of rotation and flexion of the proximal fragment utilising techniques causing minimal biological damage was considered paramount^5^. High velocity injuries in the young and low energy osteoporotic fractures in the elderly are the two main etiological factors leading to the bimodal distribution of these fractures which was observed in our study as well^5^. We found no significant association between factors like age, gender of the patient, affected side, mechanism of injury, comorbid conditions, presence of associated injuries and osteoporosis with respect to fracture healing.

Kaufer, in his study on complications observed in intertrochanteric fracture, mentions about five variables viz; quality of the bone, fracture geometry, implant selection, placement of the implants and reduction achieved as the determinants of outcome^6,11^. Since these are applicable in general for most fracture management, this can be extrapolated to subtrochanteric fractures as well. Of these, bone quality and fracture geometry are not in the treating surgeons’ control, but the implant related factors are. The intramedullary nail has high stiffness, higher load to failure, and shorter lever arm when compared to the extra-medullary devices and has become the standard implant in the treatment of subtrochanteric fractures^2,12-14^. Reducing the fractures before reaming for nailing and proper entry point are the prerequisites for appropriate placement of intramedullary devices. Entry points slightly medial to the trochanteric tip and avoiding the lateral entry are the key points for preventing varus malalignment^8^. Therefore, the quality of fracture reduction becomes the only modifiable determinant under the control of treating surgeon. Hence, we suggest a low threshold for considering either a percutaneous or mini open (in order to preserve the biology by using small footprint instruments) modality of reduction at the fracture site, to get the anatomical alignment when the closed reduction fails^4^.

In literature, there is no consensus regarding the definition of the subtrochanteric area^1,15-17^. There are more than 17 documented classification systems which are available for these fractures^6,7,16,18-20^. Evidently, none of the classifications fulfil all the requirements and is of little or no use in predicting the prognosis. We classified these fractures as high and low types depending on the majority of fracture line component with relation to the lesser trochanter which is similar to Russel Taylor type II and type I classification of subtrochanteric fractures^21^.

There was an increased occurrence of delayed or non-union observed in the low type of fractures in our study. Moreover, mean time taken for union was also increased in the low type of fractures. It cannot be disregarded that the high type of fractures occurs in a more cancellous area. Also, the presence of a lateral butterfly fragment, decreases the combined muscle force acting on the proximal fragment, which in turn leads to reduced stress at the fracture site in the high type of fractures. The blood supply is also better in high type of fractures due to more soft tissue attachments. Femoral nutrient artery usually arises from the second or third perforator which regularly gets injured in a low type of subtrochanteric fracture, further leading to disruption of the blood supply to this region^4,22^. All these anatomical and mechanical properties could have contributed to better fracture healing in the high type of subtrochanteric fractures.

In our study, when we compared fracture union among the cases with lateral or medial butterfly fragments, the presence of medial butterfly fragment is significantly associated with delayed or non-union. It is well known that the subtrochanteric region experiences the high compressive stresses which act on the medial cortex and tensile stresses act along lateral cortex^3,15,17^. Disruption of continuity in the medial cortex due to inappropriate reduction or comminution is expected to result in the transmission of the stresses through the implant and subsequent varus collapse, implant failure, screw cut out, delayed or non-union^12,13,17,23^. This emphasises the fact that medial cortical continuity is important as it is the region where the highest compressive forces are acting^9^. One interesting point to note in our study is that the majority of low type of fractures have medial butterfly fragments (Table I). With the increase in incidence of delayed and non-unions in low type of fractures, this factor highlights the need to establish medial cortical continuity in subtrochanteric fractures of this type.

It is always recommended to reduce the fracture before making an entry for the nail and reaming. Anatomical alignment and establishment of medial cortical contact has been found to improve fracture healing and hence gives better outcome^24^. Even though the opening of the fracture site disturbs the fracture hematoma and extensive periosteum stripping is discouraged; mini open techniques of using ball spike pusher, Schanz pin as a joystick, bone lever, bone clamps is desirable so as to get proper reduction^25,26^. Besides, there are no consequential complications regarding infection or union reported with mini-open techniques^27^. We didn’t find any significant difference in healing patterns in fractures treated with closed or mini open techniques in our study too. Cerclage wire is a versatile tool which has been recommended to reduce and hold the reduction^22,28,29^. In our study, cerclage wire was used in two cases without any complications.

Our study has a relatively younger cohort with high energy injuries and low comorbid conditions. Our results were comparable with similar studies in literature (Table III)^7,9,24,27,30-34^. We noted that varus malreduction in the coronal plane is associated with delayed union and non-union. Further analysis revealed the cut of value of 8.5° of coronal plane varus malalignment. To our knowledge, not many studies have quantified the tolerable varus malreduction in these fractures (Table III). Our findings are similar to Krappinger *et al*^9^. We partially agree with Riehl *et al*, who states that malreduction in the coronal or sagittal plane of more than 10° is associated with an increase in delayed or non-union^32^. We didn’t find a significant association between sagittal plane malalignment and fracture healing. One explanation for such a finding might be the fact that flexors and extensors which are acting across the proximal fragment are equally strong. On the contrary, hip abductors are much stronger when compared to the adductor component in the proximal fragment leading to more significant continuous deforming forces in the coronal plane.

**Table III:** Comparison with other previous studies.

**Study**	**No. of cases**	**Complication**	**Comments**
Krappinger *et al* (2019)^9^	73 (3 different nails)	Non-union: 17	Varus angulation and lack of medial cortical support are the risk factors.
Mingo-Robinet *et al* (2015)^24^	26	Nil	- Union time 9.65 weeks (range: 8 – 16 weeks). - Reduction before nailing is mandatory. - Minimally invasive clam reduction with cerclage wires is safe.
Georgiannos *et al* (2015)^30^	- Long gamma nail 3 (LG3N) = 75 - Long trochanteric gamma nail (LTGN) = 83	LG3N - Intra-op : 4 (5.3%) - Post-op : 9 (12%) LTGN: - Intra-op : 9 (10.8%) - Post-op : 20 (24%) Most common complication: Screw cutout	LG3N is biomechanically better when compared to LTGN.
Jiang *et al* (2018)^31^	36	Non-union: 5	- Union time 6.8 months (range: 3 – 17 months). - Fracture displacement > 2.2cms leads to nonunion.
Reihl *et al* (2014)^32^	35	- Non-union: 1 (2.9%) - Delayed Union: 13 (37%)	Malreduction >10° in coronal or sagittal plane leads to non-union and delayed union.
Beingessner *et al* (2013)^27^	96	- Non-union: 5 - Screw removal: 6	High union rate by open reduction with minimal malalignment.
Afsari *et al* (2009)^7^	44	Non-union: 1	High union rate and minimal malalignment with clamp reduction and judicious use of circlage wires.
Shukla *et al* (2007)^33^	60	- Implant failure: 9 - Non-union: 3 - Deep infection: 1	High union rate and better alignment by open reduction.
Zhou *et al* (2015)^34^	76	Delayed union: 1	- Average union time: 4.5 months. - Excellent functional recovery (Harris hip score): 65 patients - Intra-operative reduction important.
Our study	60	- Delayed union: 9 Non-union: 6 (including Implant failure: 1)	- Varus angulation >8.5° has increased incidence of non-union or delayed union. - Minimum threshold advocated for mini-open reduction to get better alignment.

We had six non-unions and nine delayed unions in our series. Of the six non-unions, one patient had varus collapse resulting in implant breakage. Another patient had neck screw back out, and subsequently non-union. We had no incidence of screw cut through and infections in our series.

Hence, as per our study, there is a higher chance of delayed or non-union in low type of subtrochanteric fractures, having medial butterfly fragment and fixed in varus malalignment with a cut off varus angulation at 8.5°. High type of subtrochanteric fractures, with or without lateral butterfly fragment, low type with lateral butterfly fragment, fixed in anatomical alignment by closed or open reduction, have better healing potential.

Adequate follow-up of a significant number of patients with data collected from well-maintained hospital records are the strengths of our study. Our study has a few limitations. It is a single centre study with no population diversity. There is no functional evaluation to correlate with the radiological outcomes. We measured the osteoporosis indirectly by quantifying the bone mineral density using cortical thickness index in lateral view^10^. We relied upon the radiographic findings to determine the union status. A multicentric prospective study with larger number of cases and functional evaluation would further validate the data.

## CONCLUSION

Subtrochanteric fracture of the femur in adults is truly a ‘difficult fracture’ to treat, despite recent advancements in fracture fixation techniques, due to the inherent unique anatomy of the region. The high type of these fractures with or without butterfly fragment and low type with a lateral butterfly fragment are noted to have a better outcome than the low type of fractures with loss of medial continuity. Varus malalignment is least tolerated in this zone. For a good outcome, it is recommended to achieve reduction primarily, within 8.5° of varus malalignment in the coronal plane either by closed or by having minimum threshold for utilising mini open techniques.

## CONFLICT OF INTEREST

The authors declare no conflicts of interest.
